# Spatio-temporal optimization of sampling for bluetongue vectors (*Culicoides*) near grazing livestock

**DOI:** 10.1186/1756-3305-6-151

**Published:** 2013-05-24

**Authors:** Carsten Kirkeby, Anders Stockmarr, René Bødker, Peter Lind

**Affiliations:** 1 National Veterinary Institute, Technical University of Denmark, Bülowsvej 27, DK-1790 Copenhagen V, Denmark; 2 Department of Applied Mathematics and Computer Science, DTU Compute, Technical University of Denmark.Matematiktorvet, Building 324, DK-2800 Lyngby, Denmark

**Keywords:** *Culicoides* obsoletus, Spatial variation, Light traps, Abundance, Bluetongue

## Abstract

**Background:**

Estimating the abundance of *Culicoides* using light traps is influenced by a large variation in abundance in time and place. This study investigates the optimal trapping strategy to estimate the abundance or presence/absence of *Culicoides* on a field with grazing animals. We used 45 light traps to sample specimens from the *Culicoides* obsoletus species complex on a 14 hectare field during 16 nights in 2009.

**Findings:**

The large number of traps and catch nights enabled us to simulate a series of samples consisting of different numbers of traps (1-15) on each night. We also varied the number of catch nights when simulating the sampling, and sampled with increasing minimum distances between traps. We used resampling to generate a distribution of different mean and median abundance in each sample. Finally, we used the hypergeometric distribution to estimate the probability of falsely detecting absence of vectors on the field. The variation in the estimated abundance decreased steeply when using up to six traps, and was less pronounced when using more traps, although no clear cutoff was found.

**Conclusions:**

Despite spatial clustering in vector abundance, we found no effect of increasing the distance between traps. We found that 18 traps were generally required to reach 90% probability of a true positive catch when sampling just one night. But when sampling over two nights the same probability level was obtained with just three traps per night. The results are useful for the design of vector monitoring programmes on fields with grazing animals.

## Findings

Estimates of the abundance and presence/absence of vectors are essential for understanding and modeling vector-borne diseases. Light trapping is the most widely used method to sample *Culicoides* and single light traps are often assumed to be representative for abundance in a large area (e.g. [1-3]). In a previous study the number of *Culicoides* trapped in 45 CDC light traps placed in a regular grid were found to vary dramatically [4]. Spatial clusters of higher abundance were found randomly on the field, causing up to 11 times higher abundance. Huge variations in abundance between catch nights was also observed [4]. This variation can potentially have a great impact on field studies where one or a few light traps are assumed to represent the mean vector abundance on a field with grazing animals such as sheep, cattle or horses which are not confined at night (e.g. [5-9]). In this paper we analyze the data from the study from a practical perspective, quantifying the impact of spatial and temporal variation and clustering on the uncertainty of the estimated vector abundance.

We used 45 battery-operated CDC 4 W light traps (http://www.johnwhock.com) on a field (length: 750 m, width: 250 m) near Vallø, Denmark, during the summer (July - September) of 2009. A detailed description of the study is given in Kirkeby et al. [4]. The traps were evenly dispersed throughout the field in 50 by 50 m grid points [4]. 260 sheep were confined to an enclosure in one end of the field during the study. Light traps turned automatically on at dusk and off at dawn and were emptied daily. Only females of the Obsoletus group were included in the dataset. For eight catch nights half of the traps were sorted and counted due to time constraints, resulting in a 100 by 100 m grid. We carried out a series of analyses to investigate the sampling variation of *Culicoides* abundance caused by light trapping. All statistical calculations were carried out in R (R 2.12.2).

A first analysis was carried out to quantify the sampling variation on a single catch night, and was repeated for all nights. For each catch night we generated 10,000 random samples using n (1-15) traps per sample, and we then calculated the mean number of vectors in each sample, resulting in a distribution of mean vector abundances for each sample size.

A second analysis was performed to investigate if a minimum distance between the traps in a sample would improve the mean abundance estimate. We resampled one to ten traps 10,000 times using two different minimum distances between the traps: 100 m and 150 m. We used the variation in mean abundance estimates to evaluate if larger distance between traps in a sample resulted in less variance. This was carried out on data from the nights of July 21^*st*^ and August 31^*st*^ where strong clusters were found on the field [4].

In a third analysis we quantified the probability of getting a false negative result (falsely detecting absence), using a given number of traps (1-20) per sample on the field. The proportion of negative traps per night, weighted with the number of analyzed traps per night, was used as the general probability for a negative trap during the whole study period. The hypergeometric distribution was then used to calculate the probability of detecting false absence in a sample consisting of n (1-20) traps. We also removed five catch nights with more than 90% negative samples and repeated the calculations with this modified dataset.

Finally, a fourth analysis was carried out to identify the number of traps necessary to detect presence of vectors when using from one to ten traps on one, two or three randomly selected nights. Using the hypergeometric distribution as above, we determined the number of traps required for detecting the presence of vectors with 90% or 95% certainty. The number of traps required to reach the certainty levels when sampling on two and three nights was calculated by exponentiating the probabilities for one catch night to the power of two and three. This procedure was also repeated with the modified dataset. For each catch night we also calculated the probability of falsely detecting absence of vectors using one to ten traps, by exponentiating the probabilities as above. No sheep were harmed in this study. Permission to move the sheep was obtained by the owners.

A total of 16 catch nights were obtained during a 46 day period in the summer of 2009, from which 4,488 females of the *C. obsoletus* group were counted (Table 1). Out of total 530 samples, 224 (42%) were negative for *C. obsoletus* and 306 (58%) were positive.

**Table 1:** Descriptive statistics for each catch night: The number of Obsoletus group specimens caught, the mean catch per trap, the number of analyzed traps, the percentage of zero-catches, the minimum catch and the maximum catch

**Date**	**20.07**	**21.07**	**27.07**	**03.08**	**04.08**	**06.08**	**17.08**	**18.08**	**21.08**	**24.08**	**25.08**	**27.08**	**28.08**	**31.08**	**03.09**	**04.09**
**Caught**	4	872	316	173	522	612	2	93	95	29	427	1086	1	253	2	1
**Mean**	0.08	19.38	14.36	6.92	11.86	13.91	0.08	2.02	4.52	1.31	18.56	72.40	0.04	5.62	0.04	0.02
**Traps**	45	45	22	25	44	44	23	46	21	22	23	15	23	45	42	45
**% Zero-catches**	91	0	5	20	0	0	91	35	14	73	0	0	96	33	95	98
**Min.**	0	2	0	0	1	2	0	0	0	0	2	24	0	0	0	0
**Max.**	1	79	68	106	79	48	1	20	18	12	58	176	1	44	1	1

**Figure 1:**
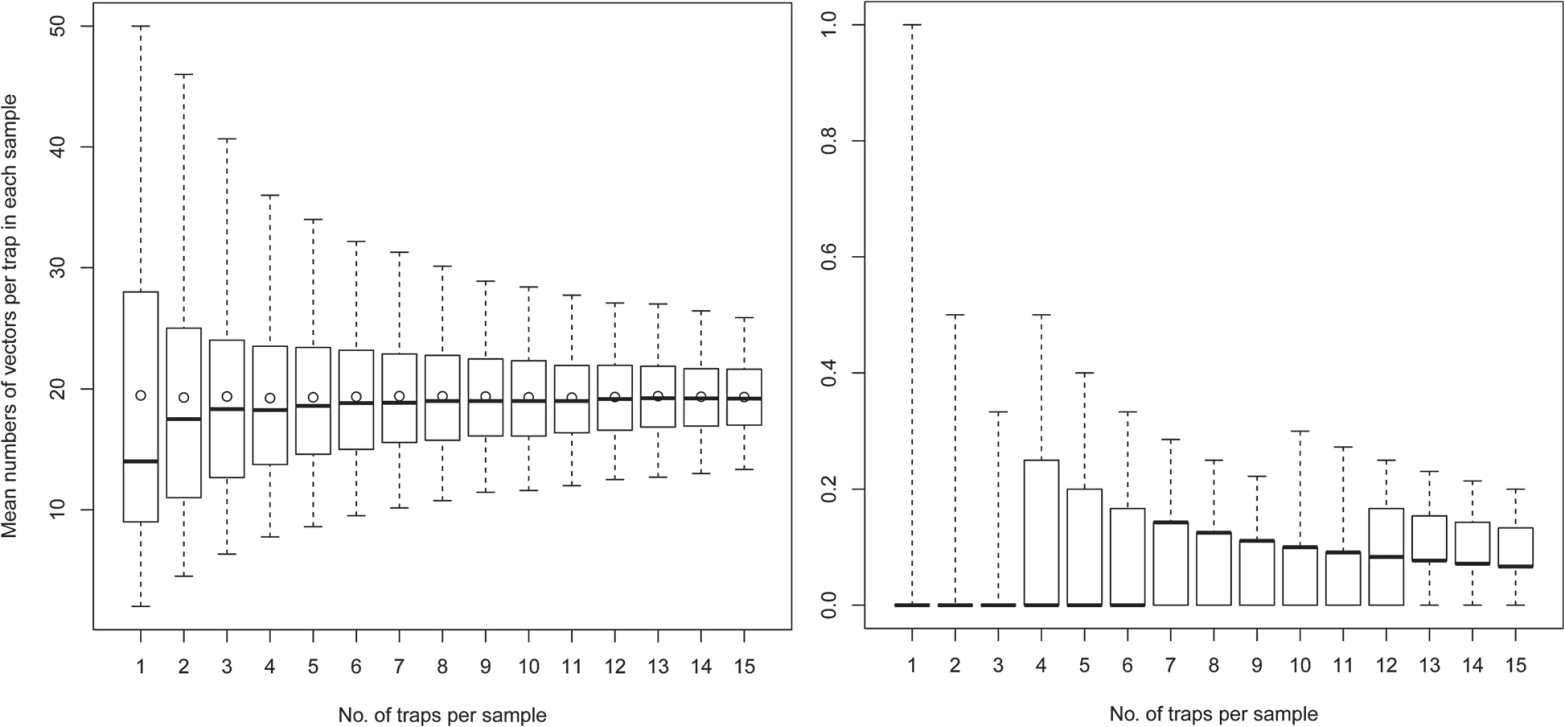
**Resampling analysis of mean catches on two selected catch nights: Left: July 20**^***th***^**, right: July 21**^***st***^**.** Circles illustrate the mean of 10,000 random samples, horizontal lines the median and whiskers show the 95% simulation envelope. Boxes show the 25% and 75% percentiles.

As expected, the vector abundance showed a declining sampling variation around the mean with an increasing sample size. This was most obvious on nights with a high total catch. An example is shown in Figure 1 (right), where the range of the 95% simulation envelope decreased from using one trap (range: 2 to 48) with 50% when using six traps (range: 10 to 30).

The second analysis examined whether a more precise abundance estimate may be obtained with a larger distance between the traps in each sample. There was only a marginal decrease in the variance of the abundance estimate when sampling with one to ten traps using a minimum 100 and 150 m distance between traps (data not shown).

**Figure 2:**
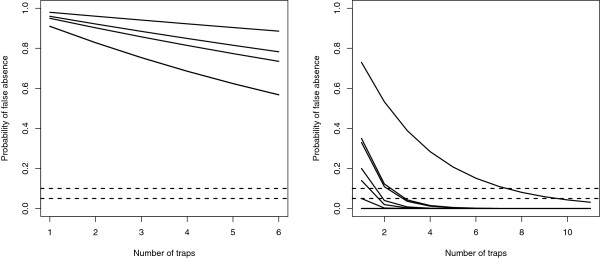
**From the field data: The probability of falsely detecting absence on the field as a function of the number of traps used for sampling.** Left: Five catch nights with more than 90% zero-catches. Right: 11 catch nights with less than 90% zero-catches. On five catch nights there was no probability of falsely detecting absence. Dotted lines show the 5% and 10% probability of falsely detecting absence of vectors.

The results of the third analysis showed a strong decrease in the average probability of falsely detecting absence when increasing the number of traps, going from 0.42 using one trap to 0.16 using ten traps per sample (Table 2). Using the modified dataset remarkably lower probabilities were found, going from 0.16 using one trap to zero using ten traps. On the five catch nights with more than 90% negative trap catches (Table 1), there was only a small effect observed when more traps were included (Figure 2 left). On the 11 catch nights with less than 90% negative trap catches, three traps were mostly necessary to reach less than 5% probability of falsely detecting absence of vectors on the field (Figure 2 right).

**Table 2:** Mean probabilities for a false negative result, depending of the number of traps

**Traps**	**1**	**2**	**3**	**4**	**5**	**6**	**7**	**8**	**9**	**10**
**Whole dataset**	0.42	0.34	0.30	0.28	0.26	0.24	0.22	0.21	0.19	0.16
Modified dataset*	0.16	0.06	0.03	0.02	0.01	0.01	0.00	0.00	0.00	0.00

^∗^The modified dataset represents the data without the five catch nights with more than 90% zero-catches.

In the fourth analysis, we calculated the number of traps required to reach less than 10% and 5% probability of finding a false negative result. Using more than one catch night remarkably reduced the necessary number of traps to reach these probabilities (Table 3).

**Table 3:** Number of traps needed to reach a certainty of 90% or 95% of excluding a false negative result when sampling one, two or three nights

**Full datasetProbability / Nights**	**1**	**2**	**3**
**90%**	18	3	1
**95%**	25	7	2
Modified dataset*			
**Probability / Nights**	1	2	3
**90%**	2	1	1
**95%**	3	1	1

With the modified dataset (i.e. without low catch nights), a higher probability level is reached much quicker. ^∗^ The modified dataset represents the data without the five catch nights with more than 90% zero-catches.

Light traps are the preferred tool for estimating *Culicoides* abundance, and often only a single trap is used. However, the large spatial variation and clustering observed in *Culicoides* abundance within a grazed field may produce large uncertainties when sampling with few traps [4]. In this study, we resampled a unique dataset to quantify this variation.

Unexpectedly, we found no effect of spatial separation of traps in a sample within the range investigated. However, the estimate of vector abundance was improved when separating the trap catches temporally, due to a large temporal variation. This yields a high probability of falsely detecting absence on some catch nights (Figure 2 left). We therefore suggest avoiding catch nights with low vector activity by using e.g. weather forecasts [10], which will increase the certainty of detecting vectors if they are present in an area.

The abundance pattern on the field was measured with CDC light traps, but the result is applicable to studies with other light traps, such as the often used Onderstepoort trap, because the mechanisms are the same. However, because the range of attraction for the Onderstepoort trap is larger than for the CDC trap, less traps may be needed to properly cover the field.

There are four main concerns about the dataset used. Firstly, there was spatial autocorrelation (clusters) in the data, and the aim of the present analysis was to address the practical implications of this effect. Secondly, we are resampling from a limited number of traps. This will cause the variation in samples with many traps to decrease more than if we were not restricted to a fixed number of traps. Thirdly, it has previously been shown that the maximum range of attraction for the CDC traps is 15.25 (12.7-18.3) m, and thus the catch areas did not overlap in this study [11]. A fourth concern is that the traps can compete with each other: specimens that are caught in one trap cannot be caught in another trap. Rigot et al. [12] investigated the competition between the more powerful Onderstepoort 8 W traps and found a statistically significant competition between the traps when placed 50 m apart, but not when placed 100 m apart. The CDC 4 W traps in this will compete less and therefore we do not consider competition a problem in the present study. Furthermore, if there was important competition in the present study, it is most likely that it would have caused a depletion of specimens in the middle of the field, which we did not find (results not shown).

To our knowledge, this is the first time that empirical data has been used to quantify the optimal sampling strategy for *Culicoides* vectors within a single field. The present study represents a worst case scenario for presence/absence studies, i.e. where vectors are present, but in low numbers and are therefore difficult to detect.

To conclude, we suggest using more than one trap for sampling a field of this size, and that the sampling is repeated over more than one catch night to obtain a more precise estimate of abundance.

## Competing interests

The authors declare that they have no competing interests.

## Authors’ contributions

This project is a part of the PhD project by Carsten Kirkeby at the Veterinary Institute at the Technical University of Denmark. Carsten Kirkeby conceived the study, carried out the planning and the field work, the sampling analyses and wrote the manuscript. René Bødker participated in the planning of the field work, analysis and discussion of the results. Anders Stockmarr participated in the planning of the field work, and took part in the analysis and the discussion of the results. Peter Lind participated in the analysis and discussion of the results. All authors read and approved the final version of the manuscript.
